# Deconvolution and IVIVC: Exploring the Role of Rate-Limiting Conditions

**DOI:** 10.1208/s12248-015-9849-y

**Published:** 2015-12-14

**Authors:** Alison Margolskee, Adam S. Darwich, Aleksandra Galetin, Amin Rostami-Hodjegan, Leon Aarons

**Affiliations:** Centre for Applied Pharmacokinetic Research, Manchester Pharmacy School, The University of Manchester, Manchester, UK; Certara, Sheffield, UK

**Keywords:** absorption, dissolution, *in vitro*-*in vivo* correlation (IVIVC), numerical deconvolution, pharmacokinetic modeling

## Abstract

*In vitro*-*in vivo* correlations (IVIVCs) play an important role in formulation development and drug approval. At the heart of IVIVC is deconvolution, the method of deriving an *in vivo* “dissolution profile” for comparison with *in vitro* dissolution data. IVIVCs are generally believed to be possible for highly permeable and highly soluble compounds with release/dissolution as the rate-limiting step. In this manuscript, we apply the traditional deconvolution methods, Wagner-Nelson and numerical deconvolution, to profiles simulated using a simplified small intestine absorption and transit model. Small intestinal transit, dissolution, and absorption rate constants are varied across a range of values approximately covering those observed in the literature. IVIVC plots and their corresponding correlation coefficients are analyzed for each combination of parameters to determine the applicability of the deconvolution methods under a range of rate-limiting conditions. For highly absorbed formulations, the correlation coefficients obtained during IVIVC are comparable for both methods and steadily decline with decreasing dissolution rate and increasing transit rate. The applicability of numerical deconvolution to IVIVC is not greatly affected by absorption rate, whereas the applicability of Wagner-Nelson falls when dissolution rate overcomes absorption rate and absorption becomes the rate-limiting step. The discrepancy between the expected and deconvolved input arises from the violation of a key assumption of deconvolution that the unknown input and unit impulse enter the system in the same location.

## INTRODUCTION

*In vitro*-*in vivo* correlations (IVIVC) play an important role in the production and approval of drug products and formulations. Once an IVIVC is established for a set of formulations, then *in vitro* dissolution tests can be used in place of further bioequivalence studies in the production or modification of different formulations (1). Thus, IVIVCs play an important role in the industry, saving time, money, and unnecessary clinical trials. At the heart of IVIVC is deconvolution, the method of deriving an *in vivo* “dissolution profile” for point by point comparison with *in vitro* dissolution data in what is termed a “level A” IVIVC by the FDA (2).

Several methods of deconvolution have been used over the past 50 years in the field of pharmacokinetics. The traditional methods, Wagner-Nelson and Loo-Reigelman, appeared in the 1960s (3,4). These methods have been used extensively to determine the absorption kinetics following an oral administration, and Wagner-Nelson is still the most frequently used method in IVIVC. Numerical deconvolution emerged in the 1970s as an alternative method of calculating drug input rates (5) (whether it be the rate of absorption or rate of dissolution).

These deconvolution methods have been applied to the calculation of IVIVCs for a variety of compounds and formulations in the literature, but often, the assumptions underlying their applicability are not discussed in detail. It has been noted in the literature that IVIVCs are generally possible for high permeability compounds and for formulations where release/dissolution is the rate-limiting step, with dissolution still occurring within the time frame of intestinal transit (6), provided that there is a dissolution method that can effectively differentiate between different release characteristics. This manuscript will further challenge this general rule and intentionally push the boundaries to reveal where deconvolution can be safely used in IVIVC, and where it can be expected to fail.

We focus on the traditional deconvolution methods, Wagner-Nelson and numerical deconvolution, and their applicability under different rate-limiting conditions. We chose the former since it is the most widely used deconvolution method in IVIVC and the latter since it can be used to predict *in vivo* dissolution separately from absorption. The sensitivity of IVIVC with these methods to different rate-limiting conditions was tested on a simplified compartmental transit model. This was achieved by generating a number of simulations, each with a different combination of absorption, dissolution, and transit rates, applying the deconvolution methods to the resulting profiles and establishing an IVIVC for each case.

## METHODS

A simplified absorption and transit model was used to test the effects of changing dissolution, absorption, and transit rates on the applicability of two deconvolution methods in establishing IVIVCs. Intestinal transit, dissolution, and absorption rates were varied across physiologically meaningful ranges obtained from the literature. For each combination of parameters, the profiles following administration of an oral solution and an immediate release formulation were simulated. Wagner-Nelson and numerical deconvolution were applied to obtain the deconvolved absorption profiles. These absorption profiles were plotted against the respective fraction dissolved curves representing the *in vitro* dissolution curves that would be used to establish an IVIVC. The corresponding correlation coefficients for each *in vitro* fraction dissolved *vs. in vivo* fraction absorbed plot were calculated in order to gauge the effect of transit, dissolution, and absorption rates on the interpretation of deconvolution. Following the analysis of the applicability of these methods to IVIVC in different situations, we analytically deconvolved absorption models of smaller size to obtain closed form solutions to the deconvolved input so that the reasons for the discrepancies between the deconvolution methods and the simulated *in vitro* dissolution profile could be determined. The equations associated with Wagner-Nelson and numerical deconvolution used in this investigation are provided in the Appendix.

## TEST MODEL

### Model Structure

**Fig. 1:**
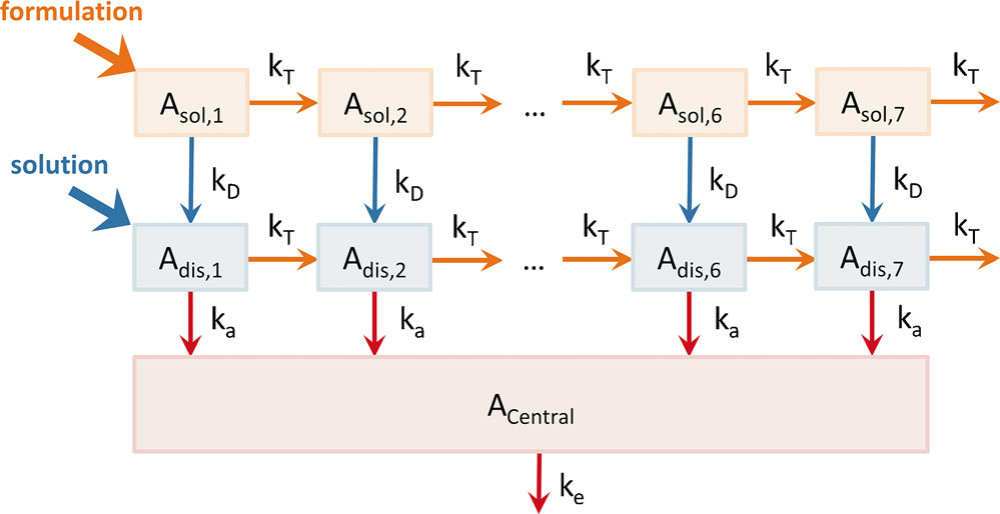
Scheme for a 7 compartment absorption and transit test model. *A*_*sol*,*i*_ and *A*_*dis*,*i*_ represent the amount of solid and dissolved drug, respectively, in compartment *i*, and *A*_*central*_ represents the amount of drug in the central compartment. Transit, dissolution, absorption, and elimination are governed by first order rate constants *k*_*T*_, *k*_*D*_, *k*_*a*_, and *k*_*e*_, respectively

In this study, we tested deconvolution on a series of simplified absorption and transit models, based on the traditional compartmental transit model (i.e., Yu *et al.* 1996) (7). The simplified model used here is comprised of seven intestinal segments, each with solid and dissolved compartments (see Fig. 1). An oral dosage starts in *A*_*sol*,1_ (or *A*_*dis*,1_ for an oral solution). The solid drug in each segment, *A*_*sol*,1_, can either transition to the next segment, *A*_*sol*,*i* + 1_, at a rate of, *k*_*T*_ ⋅ *A*_*sol*,*i*_ or dissolve according to the rate *k*_*D*_ ⋅ *A*_*sol*,*i*_ and thus enter *A*_*dis*,*i*_. From *A*_*dis*,*i*_, the dissolved drug can be absorbed into the systemic circulation at a rate of *k*_*a*_ ⋅ *A*_*dis*,*i*_. Drug is eliminated from the central compartment with rate *k*_*e*_ ⋅ *A*_*central*_. The system of differential equations describing this model is presented below.

$$ \begin{array}{l}\frac{d{A}_{sol,1}}{dt}=-\left({k}_T\cdot {A}_{sol,1}+{k}_D\cdot {A}_{sol,1}\right)\hfill \\ {}\frac{d{A}_{dis,1}}{dt}={k}_D\cdot {A}_{sol,1}-\left({k}_T+{k}_a\right)\cdot {A}_{dis,1}\hfill \\ {}\frac{d{A}_{sol,i}}{dt}={k}_T\cdot {A}_{sol,i-1}-\left({k}_T\cdot {A}_{sol,i}+{k}_D\cdot {A}_{sol,i}\right),\mathrm{f}\mathrm{o}\mathrm{r}\;i=2\dots 7\hfill \\ {}\frac{d{A}_{dis,i}}{dt}={k}_T\cdot {A}_{dis,i-1}+{k}_D\cdot {A}_{sol,i}-\left({k}_T+{k}_a\right)\cdot {A}_{dis,i},\mathrm{f}\mathrm{o}\mathrm{r}\;i=2\dots 7\hfill \\ {}\frac{d{A}_{central}}{dt}=\left({\displaystyle \sum_{i=1}^7{k}_a\cdot {A}_{dis,i}}\right)-{k}_e\cdot {A}_{central}\hfill \end{array} $$

For simplicity during our analysis, we assumed that dissolution followed a first order process with rate constant *k*_*D*_ and that this rate constant was the same for each compartment. These assumptions are not completely representative of the true physiology since the increasing pH gradient along the intestinal tract can affect the solubility and therefore the dissolution rate of formulations. In addition, the first order rate constant for absorption, *k*_*a*_, was assumed the same in each compartment. However, varied surface area of the intestinal segments, and the varied surface area of villi and microvilli along the intestinal tract (8) suggest this is also an oversimplification. Making these assumptions allowed for clear analysis of the effect of changing the dissolution and absorption rates and their impact on the interpretation of the deconvolved input. The values for these parameters and the remaining system parameters are discussed in the following section.

### Parameter Values

**Fig. 2:**
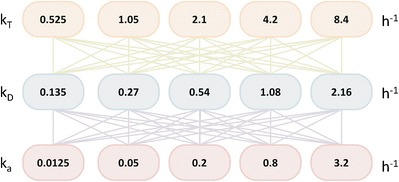
Parameter values used for analysis of the effects of intestinal transit, dissolution, and absorption (*k*_*T*_, *k*_*D*_, and *k*_*a*_, respectively) on the applicability of the traditional deconvolution methods in IVIVC. Parameter values were selected to approximately cover those observed in the literature (7,9). Simulations using the 7 compartment intestinal model were run with each combination of parameters, obtaining a set of 5 × 5 × 5 simulations

The effects of changing transit rate, dissolution rate, and absorption rates on the results of deconvolution were analyzed. The 7 compartment intestinal transit model was solved under various scenarios, each with a different set of values for these parameters, which were selected according to a range of typical values approximately covering those observed in the literature (7,9–11). Five values were selected for each of the transit, dissolution, and mean absorption rates (see Fig. 2). Simulations using the 7 compartment model were run with each combination of parameters, obtaining a set of 5 × 5 × 5 simulations (totaling 125 simulations; see Appendix Fig. 8 for the simulated plasma concentration time profiles).

A range of transit rates were calculated based on total small intestinal transit times of 50, 100, 200, 400, and 800 min, which approximately covers the range of transit times described in Yu 1996 (mean 200 min, minimum 30, maximum 570) (7). The total small intestinal transit rate constants from this range of transit times are 1.2, 0.6, 0.3, 0.15, and 0.75 h^−1^. Each simulation assumed one of these transit rates for the entire small intestine, which was adjusted accordingly for seven compartments, i.e., individual compartment transit rate constants of 8.4, 4.2, 2.1, 1.05, and 0.525 h^−1^.

A range of dissolution rates were calculated based on 96% dissolved after 24, 12, 6, 3, and 1.5 h. Assuming first order dissolution, this gives dissolution rate constants of approximately 0.135, 0.27, 0.54, 1.08, and 2.16 h^−1^.

A range of absorption rates were calculated based on effective permeability (Peff) values of 0.03 × 10^−4^, 0.12 × 10^−4^, 0.48 × 10^−4^, 1.92 × 10^−4^, and 7.68 × 10^−4^ cm/s which approximately cover the range of Peffs reported in Lennernas (2014) (minimum 0.03 × 10^−4^, maximum 8.7 × 10^−4^ cm/s) (9). Absorption rates were then calculated as 2 × Peff/*r* with a radius (*r*) of 1.75 cm (9) resulting in *k*_*a*_ values of approximately 0.0125, 0.05, 0.2, 0.8, and 3.2 h^−1^.

Median values for volume of distribution and elimination rate constants were determined from a database of compounds analyzed in the PhRMA CPCDC initiative of 2011 (10,11). The volume of the central compartment was taken to be 120 L, which is approximately the median of the PhRMA CPCDC initiative compounds reported in Jones 2011 (10) assuming a body weight of 70 kg. The elimination rate constant was taken to be 0.2 h^−1^, the median value of clearance divided by volume of distribution, calculated from the range of clearance values of the PhRMA CPCDC initiative reported in Ring 2011 (11) and the corresponding volumes of distribution for the same compound set reported in Jones 2011 (10).

After the sensitivity analysis, we analyzed the interpretation of the deconvolved output using model simulations evaluated with the mid-range values for intestinal transit rate (*k*_*T*_ of 2.1 h^−1^ for all seven compartments) and dissolution (*k*_*D*_ of 0.54 h^−1^). For the absorption rate, we took a typical value for a highly permeable compound since IVIVC is considered to be more identifiable for highly permeable compounds (6). In addition to the 7 compartmental transit model described above, 2 and 1 compartment transit models were also analyzed. For these models, transit rates were adjusted accordingly. For the parameter values used during the analysis of models of various dimensions, see Table I.

**Table I:** Parameter Values Used for Analysis of the Intestinal Models of Various Dimensions

Model	Dose (mg)	*V*_*D*_ (L)	*k*_*T*_ (h^−1^)	*k*_*D*_ (h^−1^)	*k*_*a*_ (h^−1^)
7 Compartment	200	120	2.1	0.54	3.2
2 Compartment	200	120	0.6	0.54	3.2
1 Compartment	200	120	0.3	0.54	3.2

Middle range values for volume of distribution (*V*_*D*_), systemic elimination (*k*_*e*_), intestinal transit (*k*_*T*_) and dissolution (*k*_*D*_) were selected from the literature (7,10,11). A high absorption rate was selected for in depth analysis since IVIVC is considered to be more identifiable for highly permeable compounds

### Simulation Conditions

For each parameter set, the test model was used to simulate plasma concentration profiles following administration of immediate release formulation and oral solution. The oral solution was simulated using initial condition of the total dose in the first dissolved compartment (*A*_*dis*,1_) and zero elsewhere. The immediate release formulation was simulated using an initial condition of the total dose in the first solid compartment (*A*_*sol*,1_) and zero elsewhere. The system of differential equations was solved in Matlab version R2013a (The Mathworks, Inc., Natick, Massachusetts) using the built-in ordinary differential equations solver ode45. The amount of drug in the central compartment, *A*_*central*_, was divided by *V*_*D*_ to obtain the simulated plasma concentration profiles.

## DECONVOLUTION METHODS AND IVIVC PROCEDURE

Wagner-Nelson was applied to the simulated oral formulation concentration profiles, and numerical deconvolution was applied to the oral formulation profile, using the oral solution profile as the unit impulse response (UIR) (see Appendix for more details on the application of these methods). For the elimination rate constant which is needed in the calculation of fraction absorbed (fa) *via* Wagner-Nelson, we used the actual value of *k*_*e*_ from the simulations, in order to avoid the possibility of flip-flop kinetics that can occur when *k*_*a*_ or *k*_*D*_ is the rate-limiting step. This simulates the ideal scenario in which one had the perfect intravenous bolus UIR. During numerical deconvolution, the deconvolved input for the oral formulation was assumed to take the form of a continuous piecewise quadratic with continuous first derivative (12).

Note that Wagner-Nelson relies solely upon observed drug concentrations in the central compartment, and so the result reflects all processes leading up to systemic circulation, including absorption as well as dissolution. In contrast, using an oral solution as the UIR in numerical deconvolution provides a distinction between the systemic appearance of the oral solution and the other dosage form. This difference is considered to represent *in vivo* dissolution, assuming that all processes associated with absorption of the solid formulation and oral solution are identical. Potential errors in and consequences of this assumption are discussed later in this paper.

Predicted *in vivo* fraction of drug absorbed and dissolved over time obtained through deconvolution using the Wagner-Nelson and numerical deconvolution methods, respectively, were plotted against the corresponding simulated *in vitro* dissolution profiles. The simulated *in vitro* dissolution profiles were assumed to be from an ideal *in vitro* experiment, in which the same first order kinetics were observed as those that occurred within the simulated *in vivo* system. The reason for assuming this ideal situation was to identify any discrepancy between the deconvolution methods and the dissolution profiles that they are intended to represent. Correlation coefficients were obtained for each *in vitro* fraction dissolved *vs. in vivo* fraction absorbed/dissolved plot, for each combination of parameters simulated, and each of the two deconvolution methods tested. These correlation coefficients were analyzed for trends across the different parameter values and deconvolution methods. In order for the correlation coefficients to have practical meaning, they were calculated over the time points 1, 5, 15, 30, and 45 min, and 1, 1.5, 3, 6, and 12 h, simulating realistic sampling times.

## RESULTS

### Sensitivity Analysis: Impact of Intestinal Transit, Dissolution, and Permeability on the Applicability of Deconvolution Methods During IVIVC

**Fig. 3:**
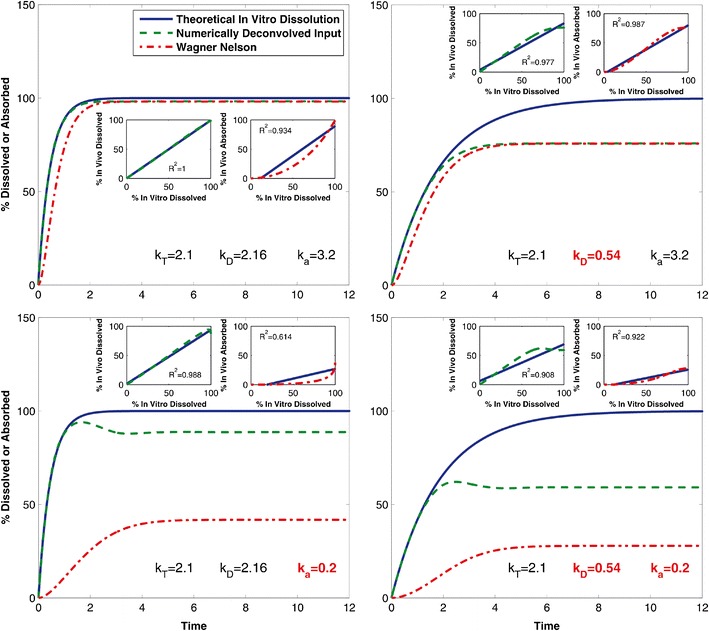
Plotted are four representative simulated *in vivo* deconvolved dissolution and absorption over time profiles obtained using numerical deconvolution (*dashed green curves*) and Wagner-Nelson (*dot*-*dashed red curves*) for different combinations of parameters, plotted against the theoretical *in vitro* dissolution profile (*solid blue curves*). Subpanels display the respective % dissolved *in vitro vs.* % dissolved *in vivo* plots for numerical deconvolution (*dashed green curves*) and % dissolved *in vitro vs.* % absorbed *in vivo* plots for Wagner-Nelson (*dot*-*dashed red curves*) beach of the two deconvolution methods, along with the associated regression lines (*solid blue curves*) and their *R*-squared values. *Panels on the left* represent the highest dissolution rate simulated, *k*_*D*_ =2.16 h^−1^, while *panels on the right* represent the middle value of *k*_*D*_ = 0.54 h^−1^. *Panels on the top* represent the highest absorption rate simulated, *k*_*a*_ = 3.2 h^−1^, while *panels on the bottom* represent the middle value of *k*_*a*_ = 0.2 h^−1^. All four panels represent the middle value of transit rate, *k*_*T*_ = 2.1 h^−1^

Figure 3 illustrates representative profiles of % absorbed (Wagner-Nelson) or dissolved (numerical deconvolution) *in vivo vs.* % dissolved *in vitro* with various combinations of values for the rate parameters. For the ideal case, with a highly absorbed and rapidly dissolved formulation, both Wagner-Nelson and numerical deconvolution perform comparably well, with *R*-squared values above 0.9 (Fig. 3, top left panel). Decreasing the dissolution rate introduces a discrepancy between the deconvolved inputs and the dissolution profile (Fig. 3, top right panel). However, IVIVC results in reasonable correlations with *R*-square values again above 0.9 for both methods. Decreasing *k*_*a*_ reveals the difference between the deconvolution methods during IVIVC. The numerically deconvolved profile more closely matches the simulated *in vitro* dissolution profile, while the fraction absorbed obtained with Wagner-Nelson is greatly affected by the decreased *k*_*a*_ (Fig. 3, bottom left panel). Adjusting for fraction absorbed does not resolve this discrepancy for the Wagner-Nelson method (*R*-squared value below 0.7), whereas the *R*-squared value for numerical deconvolution remains above 0.9. Figure 3, bottom right panel, displays the combined effect of decreasing both *k*_*a*_ and *k*_*D*_. Interestingly, the *R*-squared values for both the numerical deconvolution and Wagner-Nelson methods are above 0.9, despite the noticeable decrease in overall fraction absorbed.

**Fig. 4:**
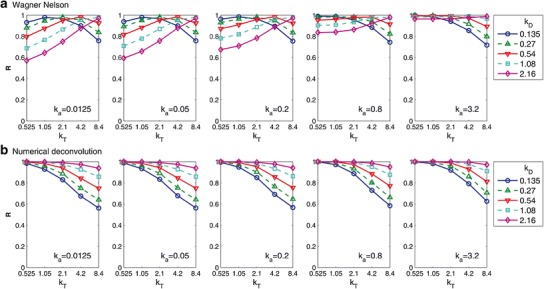
Correlation coefficients (*R*) for IVIVCs obtained using Wagner-Nelson (*top panels*) and numerical deconvolution (*bottom panels*), varying the rates for dissolution, absorption, and transit. Within each panel, transit rate constants increase from 0.525 on the *left* to 8.4 h^−1^ on the *right* and dissolution rate constants increase from 0.135 (*blue circles*) to 2.16 h^−1^ (*magenta diamonds*). Each panel represents a different absorption rate constant, increasing from 0.0125 in the *leftmost panel*, to 3.2 h^−1^ in the *rightmost panel*

Figure 4 plots the correlation coefficients for the IVIVCs obtained using the Wagner-Nelson (top panels) and numerical deconvolution (bottom panels) methods, varying *k*_*D*_, *k*_*a*_ and *k*_*T*_. It is clear from this figure that for numerical deconvolution, the highest correlation can be seen when *k*_*D*_ and *k*_*a*_ are highest, and *k*_*T*_ is the lowest (see the rightmost panel, topmost curve, leftmost point). In each panel, the correlation increases with decreasing *k*_*T*_ (following each graph from right to left), and the correlation increases with increasing *k*_*D*_ (moving from the bottommost to the topmost curve within each of the panels). Increasing *k*_*D*_ effectively decreases the transit rate within the solid compartments, decreasing the effect of transit on the numerically deconvolved input. *k*_*a*_ has little impact on the correlation obtained with numerical deconvolution.

In contrast, *k*_*a*_ has a great impact on the correlations obtained for the Wagner-Nelson method. For the highest *k*_*a*_, the relationship between the effects of transit and dissolution on the correlation mirrors that of numerical deconvolution. For low *k*_*T*_, dissolution rate has little impact on the correlation, while for high *k*_*T*_, the higher the dissolution the better the correlation. As *k*_*a*_ is decreased, an interesting relationship between transit rate and dissolution rate is revealed. For decreasing values of *k*_*T*_ (left of each panel), the higher values of *k*_*D*_ result in lower correlations, and this effect is steadily magnified as *k*_*a*_ decreases (from the rightmost to the leftmost panel). Following the panels from left to right, the correlation obtained for middle range values of *k*_*T*_ and *k*_*D*_ increases with increasing *k*_*a*_. This observation is in agreement with previous statements that IVIVC is more likely to be possible for highly permeable compounds.

### Analytical Deconvolution of Simplified Absorption Models: What is Numerical Deconvolution Really Giving Us?

In the previous section, we illustrated that the performance of IVIVC using Wagner-Nelson is highly impacted by absorption rate, while that using numerical deconvolution is relatively stable across different absorption rates, being affected more by dissolution and transit rates. The reason for this difference in the sensitivity to absorption rate is clear and should be expected based on what each method is measuring. The Wagner-Nelson method provides an estimate of amount of drug entering the systemic circulation, while numerical deconvolution (with an oral solution as the unit impulse response (UIR)) provides an estimate of amount of drug that has entered a dissolved state. Numerical deconvolution is still noticeably affected by transit and dissolution rates, and in this section, we will explore the reasons, looking at models with fewer transit compartments, and analytically deconvolving these model simulations.

The previous section compared the deconvolved fraction absorbed over time with a theoretical *in vitro* dissolution profile of the form ($\mathrm{Dose}\left(1-{e}^{-{k}_Dt}\right)$), the rate of which is ${k}_D{e}^{-{k}_Dt}$. However, this is not exactly the rate by which the oral formulation enters the point of application of the oral solution, which was the assumption required to use the oral solution as the UIR. Thus, we observed the differences between the simulated *in vitro* dissolution profile and the numerically deconvolved absorption profile.

**Fig. 5:**
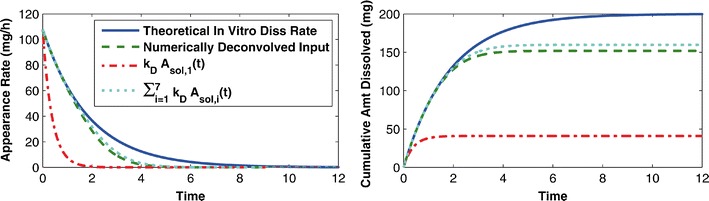
The *panel on the left* compares the numerically deconvolved input (*green dashed curve*) with the theoretical *in vitro* dissolution rate ${k}_D{e}^{-{k}_Dt}$ (*solid blue curve*), and the “reasonably expected” inputs *k*_*D*_ *A*_*sol*,1_(*t*) (*red dot*-*dashed curve*) and ∑_*i*_^7^ *k*_*D*_ *A*_*sol*,*i*_(*t*) (*cyan dotted curve*). The *panel on the right* plots the integrals over time of the “expected” and the numerically deconvolved inputs, i.e., the cumulative inputs over time. These can be thought of as the respective *in vivo* absorption/dissolution profiles which would be used to establish an IVIVC

As an alternative, it may be reasonable for one to expect the deconvolved input to be that which enters the system in the same location as the oral solution, the first dissolved compartment, namely *k*_*D*_ ⋅ *A*_*sol*,1_(*t*). However, this assumption neglects the fact that the oral formulation may travel from the first solid compartment to the next before dissolving. Keeping this in mind, another possibly more reasonable expectation for the deconvolved input may be ∑^*7*^_*i*_*k*_*D*_ ⋅ *A*_*sol*,*i*_(*t*), the sum of all of the rates by which the oral formulation enters a dissolved state. It may be hard to imagine, but this expectation is not correct either. Figure 5 illustrates the difference. The first expectation *k*_*D*_ ⋅ *A*_*sol*,1_(*t*) drastically underpredicts, while the second ∑_*i*_^7^*k*_*D*_ ⋅ *A*_*sol*,*i*_(*t*) overpredicts the deconvolved input. However, the second expectation is a vast improvement over the theoretical dissolution rate, ${k}_D{e}^{-{k}_Dt}$.

**Fig. 6:**
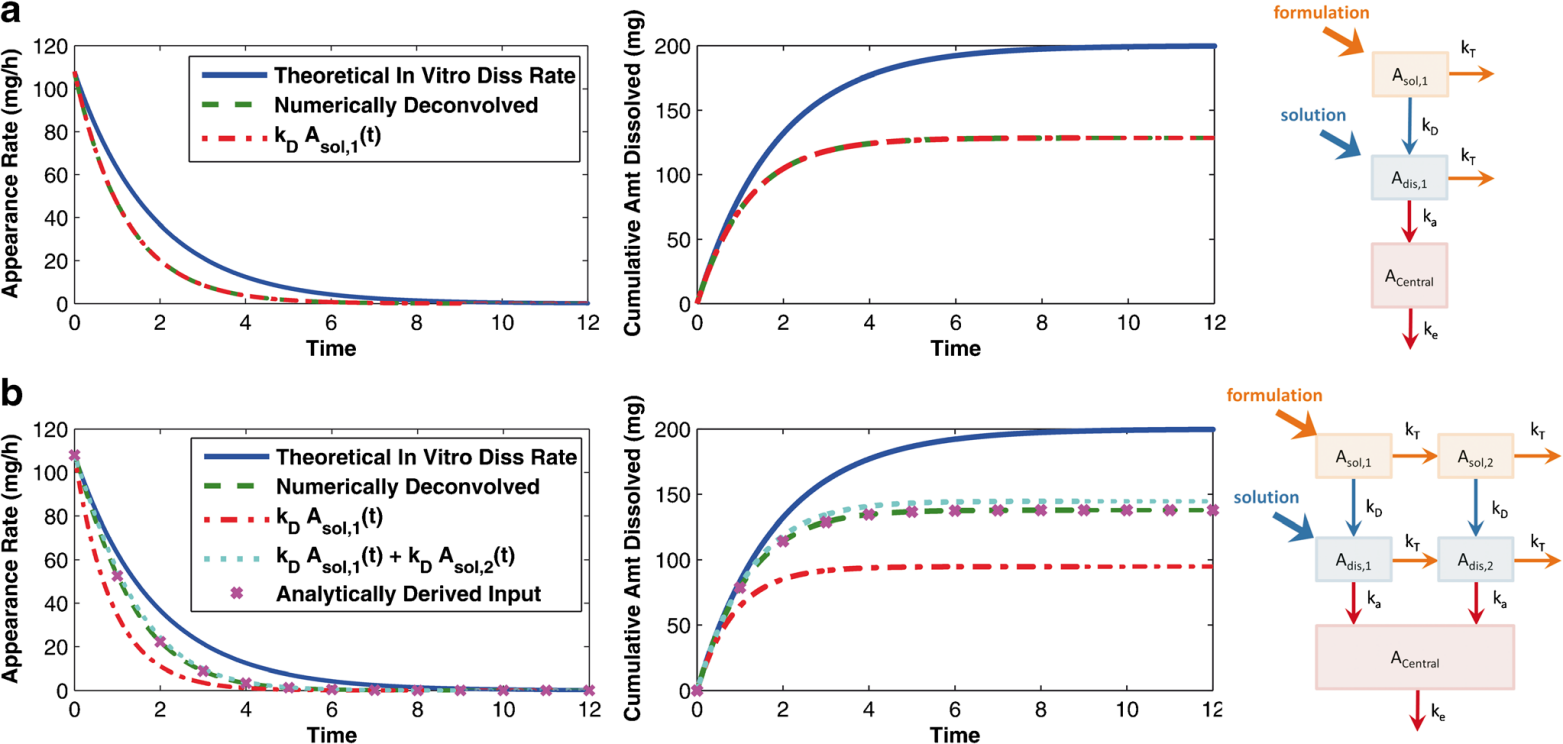
*Top left*: comparison of the “reasonably expected” input *k*_*D*_ ⋅ *A*_*sol*,1_(*t*) (*red dot*-*dashed curve*) with the numerically deconvolved input (*green dashed curve*) for the system with only one solid and one dissolved compartment (schematic diagram on the *right*). Removing intestinal transit from the model results in the numerically deconvolved input matching the “expected” input. *Bottom left*: comparison of the analytically derived input (magenta x marked curve) with the numerically deconvolved input (*green dashed curve*) and the “reasonably expected” inputs (*red dot*-*dashed and cyan dotted curves*) for the two compartmental absorption and transit model (schematic diagram on the *right*). The analytically derived input matches the numerically deconvolved input; thus, the analytical solution can be analyzed to determine why the deconvolved input deviates from the expected input. The *middle panels* represent the integral over time of the input profiles, and can be considered as the respective *in vivo* absorption/dissolution profiles

Applying the same analysis to an intestinal transit model with only one solid and one dissolved compartment reveals that the reasonably expected input, *k*_*D*_ ⋅ *A*_*sol*,1_(*t*), and the numerically deconvolved input match exactly (Fig. 6, top panels). Adding just one transit compartment to the model introduces a discrepancy between the expected and observed numerically deconvolved input (Fig. 6, bottom panels). To understand how the transit compartment affects the deconvolved input, we derived an analytical expression for the deconvolved input for the two compartment transit model.

The two compartment transit model can be solved analytically, and the deconvolved input can be determined using the properties of the Laplace transform. The analytical solution to the two compartment transit model for the oral formulation (*A*_*central*,*form*_(*t*)) in terms of that for the oral solution (*A*_*central*,*soln*_(*t*)) is

$$ {A}_{central, form}(t)={k}_D{A}_{sol,1}(t)*{A}_{central, soln}(t)+{k}_D{A}_{sol,2}(t)*{k}_a{e}^{-\left({k}_T+{k}_a\right)t}*{e}^{-{k}_et} $$

where * is the convolution operator. This expression can be easily related to the schematic diagram for the model (see Fig. 6), following the two routes by which the drug may dissolve: (1) The drug may enter the first dissolution compartment giving the first term in the above expression, and (2) the drug may transit to the second solid compartment and then to the second dissolution compartment giving the second term.

Deconvolving the oral solution from the first term in this expression is straightforward, giving *k*_*D*_*A*_*sol*,1_(*t*). Deconvolving the second term is more complex, the details of which we will omit here. The resulting analytically derived input for the two compartment transit model is

$$ I(t)={k}_D{A}_{sol,1}(t)+{k}_D{A}_{sol,2}(t)-{k}_T\left\{{e}^{-\left(2{k}_T+{k}_a\right)t}\right\}*\left\{{k}_D{A}_{sol,2}(t)\right\}. $$

Figure 6, bottom panels, plot the analytically derived input against the numerically deconvolved input, verifying that the numerical deconvolution is in agreement with the analytically derived input.

Now that we have a closed form for the deconvolved input, we can see how the transit of the solid dosage form has an impact on the form for the deconvolved input. The significance of this will depend on the magnitude of the transit rate compared to dissolution and absorption. For highly soluble and highly permeable compounds, transit will have little impact on the interpretation of the deconvolved input. For poorly soluble compounds, transit will have more of an impact. This is in agreement with the results of the previous section, where the impact of transit rate on IVIVC with numerical deconvolution was magnified for slower dissolution rates (Fig. 4b).

## DISCUSSION

Supposing it was possible to perform the perfect *in vitro* experiment that would exactly match the *in vivo* dissolution kinetics, we might assume the widely accepted deconvolution methods would be able to obtain a reasonable IVIVC. Plotting the first order dissolution curve using the simulated *in vivo* first order rate constant gives us this theoretically perfect *in vitro* dissolution profile. Plotting this simulated *in vitro* dissolution profile against the dissolution/absorption profile deconvolved from the simulated *in vivo* profiles, one can determine whether an IVIVC can be established.

The sensitivity analysis we performed offers a perspective on the rate-limiting steps involved during IVIVC. For the Wagner-Nelson method, rate-limiting transit reveals the importance of the relationship between absorption and dissolution. As *k*_*a*_ decreases, higher *k*_*D*_ gives poorer IVIVC which is in agreement with the idea that IVIVC is likely to be possible for high permeability, dissolution rate-limited formulations. However, this interaction is not observed for numerical deconvolution using an oral solution as the UIR. Numerical deconvolution gives very consistent results across the range of absorption rates, regardless of whether or not dissolution is the rate limiting step. Dissolution rate does however play a role in the applicability of numerical deconvolution to IVIVC when dissolution occurs outside of the intestinal transit window. In this case, IVIVC is more likely possible when intestinal transit is the rate-limiting step.

Though the simulated *in vitro* dissolution profile and the simulated *in vivo* dissolution incorporated into the model follow the exact same kinetics, the application of deconvolution does not obtain a reasonable IVIVC in every situation. The reason for this discrepancy is in the underlying assumptions required to apply deconvolution. Recall that there are two major assumptions in the derivation of numerical deconvolution. The first is that the system is linear, i.e., dose independent. This first assumption is not violated as all of the expressions in the test problem are linear in the state variables. The second is that the unknown input and the UIR enter the system in the same location. This is where the violation occurs.

It is clear that Wagner-Nelson is estimating the input in a location different to that where dissolution occurs since it is providing an estimate of the amount entering the systemic circulation, following both dissolution and permeation. It may not be obvious that this assumption is also violated for numerical deconvolution using the oral solution as the UIR. The transit of solid drug before dissolution is where the violation occurs.

In fact, when intestinal transit was removed from the system, and the intestinal compartments were replaced with one compartment each for the solid and dissolved drug, the deconvolved input then had the expected form of *k*_*D*_ ⋅ *A*_*sol*,1_(*t*). Including intestinal transit of the solid compartment introduces a complexity into the model so that the deconvolved input no longer has the “reasonably expected” form.

The underperformance of Wagner-Nelson for low permeability compounds should be expected based on the fact that it is measuring the *in vivo* fraction absorbed, not the fraction dissolved. The relative stability of numerical deconvolution in response to a wide range of absorption rates should also be expected since using an IR solution as the UIR measures the *in vivo* fraction dissolved. Despite this fact, Wagner-Nelson is still the method of choice in the literature. In a literature search of journal articles in 2013 in which dissolution IVIVIC was performed, Wagner-Nelson was the most frequently applied method. Of the 27 articles identified in the search, 15 applied Wagner-Nelson (e.g., 13–15), while only 4 applied numerical deconvolution (e.g., 16). The remaining either applied a different method to obtain a level A IVIVC (e.g., a mechanistic model) or did not attempt a level A IVIVC at all. Considering the class of compounds that were investigated, it is perhaps not surprising that Wagner-Nelson was generally found to be sufficient. BCS class could be identified for 13 of the 27 publications examined, and all but one of these fell into BCS class 1 or 2, indicating that they were high permeability compounds. The remaining compound was of BCS class 3, and in that investigation, a level A IVIVC was not attempted (17). The absence of level A IVIVCs in the literature for these classes of compounds might reflect on the ability (or inability) to establish such IVIVCs, as unsuccessful IVIVCs tend not to be published. Since Wagner-Nelson, as the predominant method applied in the literature, performs poorly under absorption limited conditions, it would be interesting to see if a more widespread adoption of numerical deconvolution resulted in more successful IVIVCs for class 3 and 4.

While numerical deconvolution seemed to perform consistently well across the different scenarios, this performance can be dependent upon the accuracy of the numerical method applied. In the previous section, we noted that for a smaller test problem, our numerically deconvolved input exactly matched the analytically derived input (Fig. 6b). This may not be representative of the practical application of numerical deconvolution, which can be highly sensitive to noisy data, and results can vary depending on the prescribed form for the unknown input function. So in practice, numerical deconvolution may also have its limitations.

The simplicity of our test model and the range of parameter values selected may also indicate some limitations to our analysis, as some of the simulated scenarios may not have practical significance. For example, IVIVC is mainly applied to modified release formulations which often target the colon as the site of absorption, while the test model only included the small intestine. Several of the simulated combinations may result in a slower dissolution rate than could be realistically simulated with this test model. For the simulations involving the middle k_T_ value, the residence time was 3.33 h, and a formulation that would ensure 80% dissolution in this time frame would need a k_D_ greater than approximately 0.483 h^−1^. For the longest simulated residence time of 13.3 h, k_D_ would need to be greater than 0.121 h^−1^, and for the shortest time of 0.833 h, k_D_ would need to be greater than 1.93 h^−1^. In this range, numerical deconvolution performs very well, producing correlation coefficients consistently above 0.9.

However, the simplicity of our test model may also underestimate the disparity between *in vitro* dissolution tests and the simulated *in vivo* environment since the simulations have not taken into account that dissolution may vary with pH, which varies along the intestinal tract, and that absorption may vary with surface area of the different intestinal segments. Additionally, intestinal absorption may create sink conditions *in vivo* which can increase the *in vivo* release/dissolution rate.

Experimentalists have acknowledged the discrepancy between *in vitro* dissolution testing and the *in vivo* intestinal environment. For example, some have introduced a permeability compartment into their *in vitro* dissolution methods (18), acknowledging the effect of absorption on dissolution testing. Other examples include a modified USP 2 apparatus with a built-in pH gradient to account for pH-dependent solubility of poorly soluble basic drugs (19), or a multi-compartment stomach-duodenum model to simulate the dynamic conditions brought on by transport from the stomach to the duodenum as well as pH-dependent solubility (20). Taking things even further are *in vitro* digestion models with compartments for each of the stomach, duodenum, jejunum, and ileum, with lipases and acids incorporated in the stomach and pancreatic juices and bile in a duodenal compartment, among other simulated physiological attributes (21). However, these *in vitro* methods may be difficult to translate to the *in vivo* situation, with the scaling of *in vitro* compartment volumes up to physiological volumes, the transits between the *in vitro* compartments to physiological transit times, and the effect of absorption on dissolution kinetics.

Instead of creating an all encompassing *in vitro* system, physiologically based *in silico* models (e.g., GastroPlus™, PK-Sim®, and SimCYP®) have gone a different route by taking separate dissolution and absorption measurements *in vitro* and physiological parameters obtained *in vivo* and combining them into one dynamic model within the *in silico* environment. For example, a solubility model can be fit to data obtained at a range of pH, Caco-2 permeability can be scaled to *in vivo* permeability, and these properties and more can be incorporated into an *in silico* model which accounts for the varied intestinal environment with the transition through different pH values, surface area to volume ratios, all with the appropriate transit times (22). These models have been used recently in the establishment of IVIVCs, among other applications. For example, GastroPlus™ proved useful in the case of a manufacturing site change for a low solubility compound that dissolved rapidly in the stomach, with two formulations showing similarity in dissolution at pH 2, however failing to show similarity at higher pH (23). In another application of physiologically based IVIVC, SimCYP® was useful in separately accounting for intestinal and hepatic first pass metabolism during IVIVC, which clouded the *in vivo* dissolution profile obtained with traditional deconvolution methods (24). However, these *in silico* models also have their challenges with the multitude of *in vitro* and *in vivo* data required to build and validate them (GastroPlus™: Simulations Plus, Inc, Lancaster, CA http://www.simulations-plus.com, PK-Sim®: Bayer Technology Services, Leverkusen, Germany, http://www.systems-biology.com/products/pk-sim.html, SimCYP®: Simcyp Ltd, Sheffield, UK, http://www.simcyp.com).

With experimentalists pushing the boundary of *in vitro* dissolution and absorption systems, and modellers broadening the scope of *in silico* models, it is clear that deconvolution in IVIVC does not exist in isolation. The limitations and boundaries of applicability for deconvolution that have been discussed here may well be overcome with the right choice of *in vitro* experiment or *in silico* model.

## CONCLUSION

The performance of both the Wagner-Nelson and numerical deconvolution methods during IVIVC is comparable for highly absorbed formulations, steadily declining with decreasing dissolution rate and increasing transit rate. The applicability of numerical deconvolution to IVIVC is not greatly affected by absorption rate, whereas the performance of Wagner-Nelson declines when absorption is the rate-limiting step. The theory of deconvolution relies on the underlying assumption that the unknown input and unit impulse enter the system in the same location, which is violated for slow release formulations. In order to broaden the scope of IVIVC, we must bridge the gap between *in vitro* dissolution and *in vivo* response, pushing the *in vitro* and in silico closer to *in vivo* conditions.

## Figures and Tables

**Fig. 7:**
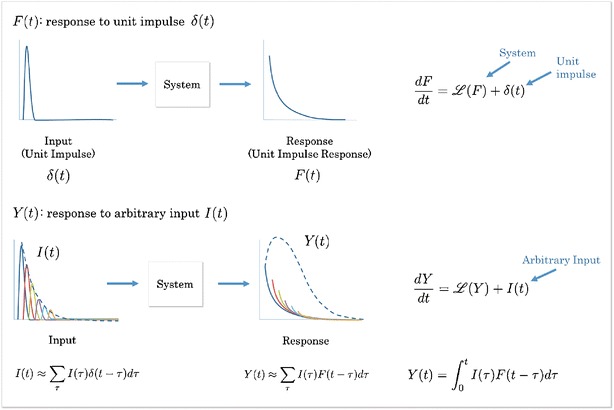
The response *Y*(*t*) of a linear time invariant system  to an arbitrary input *I*(*t*) can be approximated as the sum of responses to impulse functions *δ*(*t* − *τ*) of magnitude *I*(*τ*), for *τ* up to time *t*

**Fig. 8:**
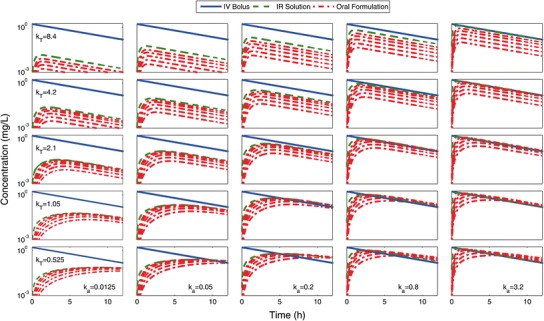
Typical plasma concentration *vs* time profiles obtained during the simulations. Each panel represents a different pairing of k_T_ and k_a_ value, with k_a_ increasing from the leftmost to rightmost panel and k_T_ increasing from the bottom- to top-most panel. Within each panel are concentration profiles following administration of an IV bolus dose (*blue solid curves*), an IR solution (*green dashed curves*), and oral formulation with the five different values for k_D_ (*red dot*-*dashed curves*)

## APPENDIX

### Linear Systems Theory

The concept of deconvolution comes out of the theory of linear time invariant systems. Deconvolution is the process of determining an unknown input rate into a system given knowledge of the response to this input, and some additional information about how the system behaves. This additional information is the response of the system to a unit impulse rate that is an impulse rate function, e.g., *δ*(*t*), with area under the curve equal to unity. Since the input into the system represents a rate, the area under the curve of an input rate is the total amount entering the system with this input. The unit impulse can be thought of as a very rapid injection of unit dose into the system. The response to the unit impulse is called the unit impulse response (UIR) (25); here we will use *F*(*t*) to denote this response.

An arbitrary input *I*(*t*) can be approximated as a sum of impulses *I*(*τ*)*δ*(*t* − *τ*), centered at time *τ*, with magnitude *I*(*τ*), i.e.,

$$ I(t)\approx {\displaystyle \sum_{\tau }I\left(\tau \right)}\delta \left(t-\tau \right)d\tau . $$

Assuming the system is linear and time invariant, the laws of superposition allow us to say that the response to a sum of impulses is equal to the sum of the responses to the individual impulses. Thus, the response, *Y*(*t*), to an arbitrary input *I*(t), can be approximated as a sum of unit impulse responses *F*(*t* − *τ*), centered at time *τ* with magnitude *I*(*τ*), i.e.,

$$ Y(t)\approx {\displaystyle \sum_{\tau }I\left(\tau \right)}F\left(t-\tau \right)d\tau . $$

Taking the limit of this approximation as the width of the impulses goes to zero, we have an integral expression for *Y*(*t*):

$$ Y(t)={\displaystyle {\int}_0^tI\left(\tau \right)F\left(t-\tau \right)d\tau .} $$

This integral expression is called a convolution, and is sometimes expressed using the symbol *, i.e.,

$$ I(t)*F(t):={\displaystyle {\int}_0^tI\left(\tau \right)F\left(t-\tau \right)d\tau .} $$

For a graphical interpretation of the above formulation, see Fig. 7.

If two of the three functions, *Y*(*t*), *I*(*t*), *F*(*t*), are known, then the third is uniquely defined, and can be determined. Here, we focus on the situation in which the UIR, *F*(*t*) and the response *Y*(*t*) to an unknown input *I*(*t*) are known, and the unknown input *I*(*t*) is to be determined. The process of determining an unknown input provided its response and the UIR are known is called deconvolution. In IVIVC of an oral formulation, the UIR can be the plasma concentration profile following an IV bolus dose or following an immediate release formulation or solution, and the unknown input is the rate of *in vivo* absorption/dissolution. Integrating the input over time will give the cumulative amount absorbed or dissolved, which can then be used along with the *in vitro* dissolution profile to establish an IVIVC.

### Numerical Deconvolution

Numerical deconvolution utilizes linear systems theory, applying a minimization process directly to the convolution integral to obtain the unknown input. A functional form for the input *I*(*t*) is assumed; the parameters of which can be obtained by fitting the convolved response to observed data. This optimization problem is usually performed by minimizing the sum of square residuals (SSR) between the estimated (convolved) and observed responses (25,26).

$$ \begin{array}{l}{Y}_{est}(t)={\displaystyle {\int}_0^t{I}_{est}\left(\tau \right)F\left(t-\tau \right)d\tau}\hfill \\ {}SSR={\displaystyle \sum_i{\left({Y}_{obs}\left({t}_i\right)-{Y}_{est}\left({t}_i\right)\right)}^2}\hfill \end{array} $$

Numerical deconvolution is a model invariant method, making no assumption about the mathematical form for the system, i.e., it does not assume first order kinetics for elimination, or any compartmental model, etc. Numerical deconvolution is also more flexible in the assumptions for the absorption, the flexibility of which depends on the parameterized form for the input function. When the general behavior of the absorption kinetics is known *a priori*, this can help with the choice of the form for the input function, e.g., a first order absorption function can be assumed if first order absorption is expected. However, a more general input function may be selected for situations where the absorption behavior is not well characterized ahead of time, or when dealing with some of the nonstandard formulations. Gamel *et al.* (12) examined several options for the input function, including a single polynomial fit, a continuous piecewise linear function, and a continuous piecewise quadratic with continuous first derivative. Numerical deconvolution is one of the more stable methods of deconvolution (12), especially when it comes to noisy data (5), much more stable than the Fourier transform method for example.

### Wagner-Nelson

The Wagner-Nelson method gives an estimate of the percent of drug absorbed as a function of time. The percent of drug absorbed at time *t* is equal to the amount absorbed up to time *t*, divided by the total amount eventually absorbed. The amount of drug that has been absorbed up to time *t* can be expressed as the amount in the body at time *t* plus the amount that has been eliminated up to that time point. Assuming first order kinetics, we have

$$ \%\;\mathrm{absorbed}=\frac{A(t)}{A\left(\infty \right)}\times 100=\frac{A(t)+k{\displaystyle {\int}_{\tau =0}^tA\left(\tau \right)d\tau }}{k{\displaystyle {\int}_{\tau =0}^{\infty }A\left(\tau \right)d\tau }}\times 100 $$

Where *A*(*t*) is the amount of drug in the system at time *t* and *k* is the first order elimination rate constant (4). If a fixed volume of distribution is assumed, amount can be replaced with concentration *C*(*t*).

$$ \%\;\mathrm{absorbed}=\frac{C(t)+k{\displaystyle {\int}_{\tau =0}^tC\left(\tau \right)d\tau }}{k{\displaystyle {\int}_{\tau =0}^{\infty }C\left(\tau \right)d\tau }}\times 100 $$

Ideally, the elimination rate constant should be estimated by fitting a one compartment model to IV plasma data. In practice, many people will use the elimination rate constant obtained from a compartmental model fit to oral data. This practice is not always adequate considering the well-known phenomenon of flip-flop kinetics which occurs when absorption is the rate-limiting step. Absorption will be the rate-limiting step for many modified release formulations, i.e., for extended or delayed release formulations which are designed to slow the absorption rate. Absorption will also be rate-limiting for poorly permeable compounds. Thus, estimating the systemic elimination rate constant from a modified release profile, or any oral profile for a poorly absorbed compound, should be avoided.

### Typical Concentration Profiles Obtained in the Simulations

Figure 8 displays typical plasma concentration *vs* time profiles following IV, oral solution, and IR formulation administration, simulated using the 7 compartment absorption and transit model for different values of *k*_*a’*_*k*_*D’*_*k*_*T*_.
